# Supplementary material for: The genome sequence of the Small Seraphim, Pterapherapteryx sexalata (Retzius, 1783) (Lepidoptera: Geometridae)
Source: Wellcome Open Res. 2026 Aug 21;11:540. [Version 1] doi: 10.12688/wellcomeopenres.27368.1 (PMC13673082; doi:10.12688/wellcomeopenres.27368.1)
Supplement: Peer review history

# Reviewer report: Reviewer response for version 1

**DOI:** 10.21956/wellcomeopenres.30123.r167362

**Published:** 2026-10-15

**Authors:** Chelsea Skojec

**Affiliations:** 1 Biology, Binghamton University, Binghamton, New York, USA

The authors present a chromosome level genome assembly for *Pterapherapteryx sexalata*, the Small Seraphim, as part of the Darwin Tree of Life project. The manuscript is well organized, and the methods are described in sufficient detail. The assembly appears to be of high quality, with 96.0% BUSCO completeness and a QV of 64.1 for haplotype 1. The sequencing and assembly approaches are appropriate, and the data are publicly available.

 Overall, I find this to be a useful genomic resource for Lepidoptera research. I have a few minor comments that I believe would improve the manuscript. The identification of a putative supernumerary B chromosome is particularly interesting. While the authors report elevated coverage and the presence of multiple copies of some B1 regions, it would be helpful to provide further explanation of the evidence supporting its classification as a B chromosome. Additionally, the estimated genome size from GenomeScope (260.88 Mb) is somewhat smaller than the final haplotype 1 assembly (289.12 Mb). A brief discussion of this difference, particularly in relation to repetitive regions or the putative B chromosome, would be useful. Although the authors provide QV and k-mer completeness estimates for both haplotypes, additional assembly statistics for haplotype 2, particularly BUSCO completeness and scaffold continuity, would allow for a more complete evaluation of the assembly. The authors also describe several manual corrections, including breaks, joins, and the removal of haplotypic duplications. It would be helpful to clarify whether these corrections were applied to both haplotypes and primarily to haplotype 1.

Are sufficient details of methods and materials provided to allow replication by others?

Yes

Is the rationale for creating the dataset(s) clearly described?

Yes

Are the datasets clearly presented in a useable and accessible format?

Yes

Are the protocols appropriate and is the work technically sound?

Yes

Reviewer Expertise:

My research focuses on insect evolution, particularly in Lepidoptera. I use molecular phylogenetics, genomics, and machine learning to study the evolution of anti-predator defenses, including mimicry and eyespots.

I confirm that I have read this submission and believe that I have an appropriate level of expertise to confirm that it is of an acceptable scientific standard.

# Reviewer report: Reviewer response for version 1

**DOI:** 10.21956/wellcomeopenres.30123.r166584

**Published:** 2026-09-18

**Authors:** Temitope Opeyemi Oriowo

**Affiliations:** 1 Leibniz Institute for the Analysis of Biodiversity Change, Bonn, Germany

Overall, a nice written introduction to the species,  however, I was wondering if a bit more words were used to explain its specific ecological role - The methods are standard and generally accepted analyses for genome assembly quality, but maybe a couple of other k-mer values should have been tested - This is a well-written genome note and a wonderful addition to the genomic resource collection. Links to the reported files also work as expected

Are sufficient details of methods and materials provided to allow replication by others?

Yes

Is the rationale for creating the dataset(s) clearly described?

Yes

Are the datasets clearly presented in a useable and accessible format?

Yes

Are the protocols appropriate and is the work technically sound?

Yes

Reviewer Expertise:

genomics, genome assembly, genome annotation, population genomics

I confirm that I have read this submission and believe that I have an appropriate level of expertise to confirm that it is of an acceptable scientific standard.
